# Telomere-to-telomere genome assembly of the goose Anser cygnoides

**DOI:** 10.1038/s41597-024-03567-8

**Published:** 2024-07-07

**Authors:** Hongchang Zhao, Hao Zhou, Guobo Sun, Biao Dong, Wenqi Zhu, Xiaohui Mu, Xiaoming Li, Jun Wang, Mengli Zhao, Wenhao Yang, Gansheng Zhang, Rongchao Ji, Tuoyu Geng, Daoqing Gong, He Meng, Jian Wang

**Affiliations:** 1 Jiangsu Agri-animal Husbandry Vocational College, Taizhou, 225300 China; 2 National Waterfowl of gene pool, Taizhou, 225511 China; 3 Key Laboratory of Veterinary Biotechnology, School of Agriculture and Biology, Shanghai Jiao Tong University, Shanghai, 201100 China; 4 Yellow Sea Fisheries Research Institute, Chinese Academy of Fishery Sciences, Qingdao, 266071 China; 5 Taizhou Fengda Agriculture and Animal Husbandry Technology Co., Ltd, Taizhou, 225511 China; 6 College of Animal Science and Technology, Yangzhou University, Yangzhou, 225000 China

**Keywords:** Genomics, Genome

## Abstract

Our study presents the assembly of a high-quality Taihu goose genome at the Telomere-to-Telomere (T2T) level. By employing advanced sequencing technologies, including Pacific Biosciences HiFi reads, Oxford Nanopore long reads, Illumina short reads, and chromatin conformation capture (Hi-C), we achieved an exceptional assembly. The T2T assembly encompasses a total length of 1,197,991,206 bp, with contigs N50 reaching 33,928,929 bp and scaffold N50 attaining 81,007,908 bp. It consists of 73 scaffolds, including 38 autosomes and one pair of Z/W sex chromosomes. Importantly, 33 autosomes were assembled without any gap, resulting in a contiguous representation. Furthermore, gene annotation efforts identified 34,898 genes, including 436,162 RNA transcripts, encompassing 806,158 exons, 743,910 introns, 651,148 coding sequences (CDS), and 135,622 untranslated regions (UTR). The T2T-level chromosome-scale goose genome assembly provides a vital foundation for future genetic improvement and understanding the genetic mechanisms underlying important traits in geese.

## Background & Summary

The domestic goose (*Anser cygnoides domesticus*) holds significant economic value as a poultry species that is extensively raised for its meat, eggs, and ornamental purposes. It boasts a rich history of domestication that dates back over 6000 years, establishing geese as one of the earliest domesticated poultry species. Geese possess distinctive biological characteristics, such as rapid growth, robust disease resistance, and livers that exhibit exception tolerance to fat accumulation, enabling them to adapt to coarse feed materials. Additionally, geese manifest unique genomic traits, including a reduced susceptibility to certain avian viruses and a remarkable capacity for lipid accumulation in the liver. These distinctive traits offer valuable insights into the study of human lipid metabolism disorders. Genomic research has emerged as a pivotal tool in unravelling the genetic characteristics and biological functions of geese. Recent advancements in genome sequencing techniques have paved the way for an in-depth exploration of the genomic structure and functional attributes of geese.

Over the years, scientists have been diligently working towards the assembly of an ideal, high-quality genome for geese. In 2015, Lu *et al*. conducted the sequencing and assembly of the goose genome using second-generation sequencing data^1^. For assembly, they employed the SOAPdenovo software^2^, resulting in a draft genome size of 1.12 Gb. The draft genome consisted of 1,049 scaffolds, with a scaffold N50 of 5.2 Mb. Subsequently, in 2016, Gao published the genome sequence map of a female Sichuan White Goose. Through re-sequencing the ancestor species, the Swan Goose (*Anser cygnoides*), they unveiled that the divergence between these two species occurred approximately 3.4–6.3 million years ago^3^. Furthermore, in 2020, a chromosome-scale genome of the goose was released. Researchers unveiled a 1.11 Gb Tianfu Goose genome with a Contig N50 of 1.85 Mb and a Scaffold N50 of 33.12 Mb^4^. In recent years, high-quality chromosome-scale reference genomes of other goose breeds, such as Xingguo gray goose^5^ and Shitou goose^6^, have been successively published. These publications have provided genetic resources and established a robust foundation for breeding and biological research in geese.

However, previous genome assemblies for geese have been hindered by the presence of numerous gaps, particularly in regions rich in repetitive sequence such as telomeres and centromeres. These regions contain crucial genetic information but are challenging to accurately sequence and assemble due to their highly repetitive nature. Telomeres and centromeric DNA sequences predominantly consist of satellite DNA and are known to evolve rapidly in eukaryotic genomes^7–9^. Advancements in sequencing technologies have paved the way for the utilization of long-read technologies such as Oxford Nanopore (ONT) and Pacific Biosciences’ high-fidelity (HiFi) sequencing to address these challenges and fill gaps in animal and plant genomes. Notably, a recent breakthrough in genomics research led to the development of a Telomere-to-Telomere (T2T) assembly of human^10^ and the domestic chicken^11^ genome, successfully bridging most of the gaps in the previous assembly and providing valuable insights into the structural characteristics of telomeres and centromeres. However, a comprehensive, gapless reference genome for geese is currently unavailable. In this study, we present the first gapless assembly of the goose genome, employing various assembly strategies and leveraging high coverage and accurate long-read sequencing data. This assembly significantly enhance our understanding of the structural features of highly repetitive regions, including telomeres and centromeres, in geese. Consequently, it established a solid foundation for further investigations into the genomic structure and functionality of geese.

**Fig. 1:**
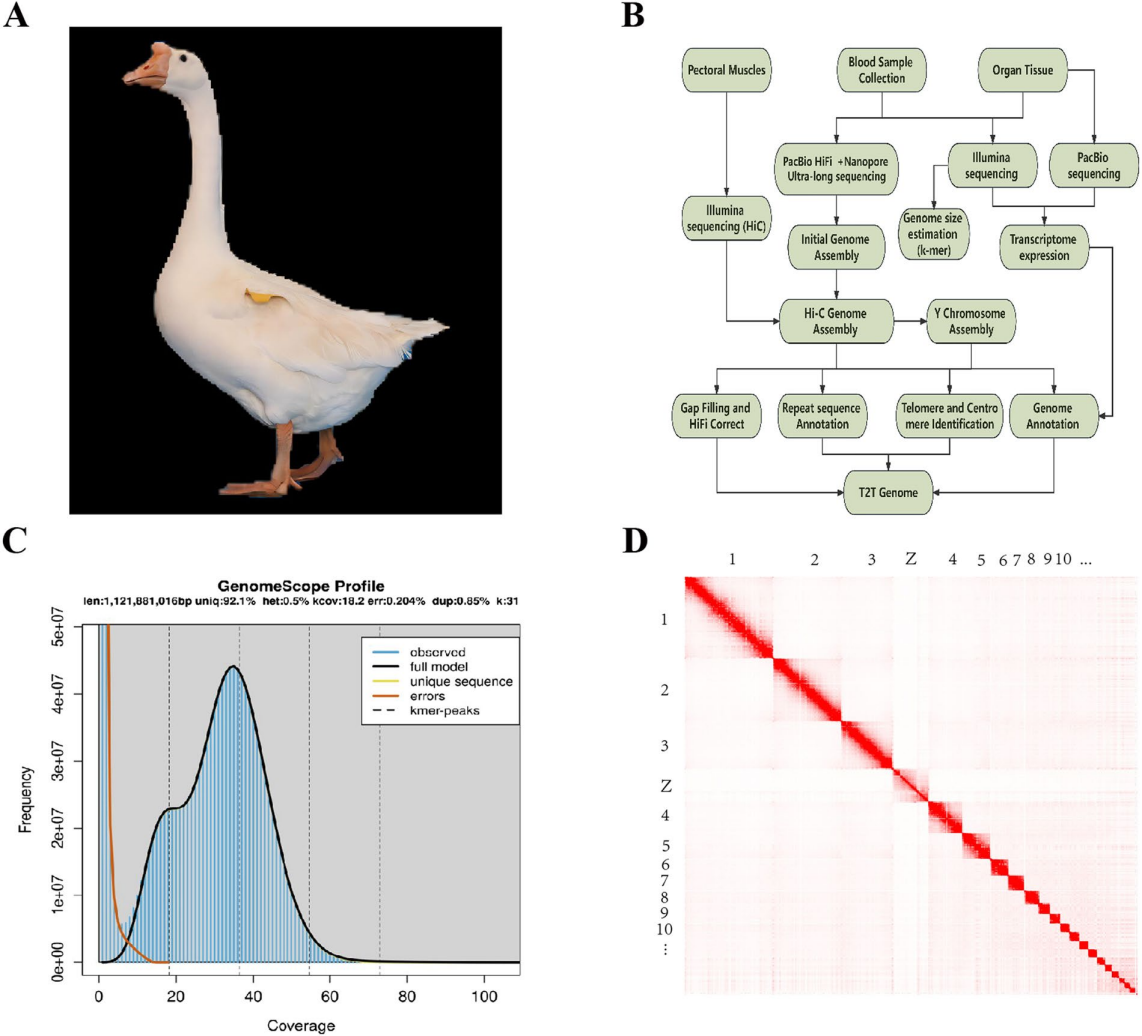
Sample photographs and genomic quality assessment charts. (**A**) Morphological photograph of Taihu goose. (**B**) Genome assembly technology route. (**C**) Genome size estimation by Genome Scope2. (**D**) Genome- wide Hi-C heatmap of Taihu goose.

In this study, we selected the Taihu goose, an exemplary local breed in China, as our research subject (Fig. 1A). By utilizing advanced techniques such as Pacific Biosciences HiFi reads, nanopore long reads, Illumina short reads, and chromatin conformation capture (Hi-C), we successfully achieved a Telomere-to-Telomere (T2T) level assembly of the goose genome (Fig. 1B). This newly assembled T2T goose genome significantly bridges the gaps present in the existing reference genome for geese. The assembled genome has a total length of 1,197,991,206 bp, with a scaffold N50 value of 81,007,908 bp. Our assembly consists of 73 scaffolds, including 38 autosomes and one pair of Z/W sex chromosomes. Importantly, 33 autosomes have reached a completely gapless state. The completion of the T2T-level goose genome provides a robust foundation for future investigations in goose functional genomics and facilitates breeding enhancements targeting economically important traits.

## Methods

### Sample collection and sequencing

#### Collection and extraction of DNA and RNA samples

The research samples were obtained from an adult female Taihu goose belonging to the conservation population of the National Waterfowl Gene Bank in Jiangsu, China. A comprehensive sequencing strategy involving multiple techniques was employed for the assembly of the goose genome. Prior to the slaughter of the goose, blood samples were collected from the wing vein using a 5 ml anticoagulant blood collection tube (BD Vacutainer® EDTA). DNA extraction was subsequently performed on the blood samples to facilitate further sequencing analysis. To obtain complete genome fragments, a combination of third-generation long-read sequencing and second-generation sequencing methods was utilized. Additionally, pectoral muscle tissue samples were dissected into small pieces and subjected to a formaldehyde cross-linking reaction for Hi-C library preparation and subsequent sequencing. Moreover, various tissues including brain, heart, liver, spleen, lung, and kidney were collected. Each tissue sample was cut into small pieces and placed in 1.8 ml cryotubes (Nunc Cryo Tube). The samples were rapidly frozen in liquid nitrogen and temporarily stored at −80 °C in an ultra-low temperature freezer (Hair DW-86L728J) for second-generation transcriptome sequencing. Furthermore, to enhance the accuracy of gene annotation, equal amounts of the six tissue samples were mixed and subjected to third-generation full-length transcriptome sequencing. All sampling procedures described above strictly adhered to the regulations set by the Animal Welfare Committee at Jiangsu College of Agriculture and Animal Husbandry Technology (22110313195050999).

The DNA extraction process from the samples strictly followed the protocols outlined in the Tiangen Blood/Cell/Tissue Genomic DNA Extraction Kit (TIANGEN® DP304). After DNA extraction, the quality of the DNA was assessed using a Nanodrop 2000 spectrophotometer. The acceptable parameters for sample DNA quality were defined as an OD ratio (260/280) ranging from 1.8 to 2.0 and a concentration exceeding 100 ng/μl. Subsequently, prepared 2% agarose gel electrophoresis was conducted to verify the integrity of the DNA samples, ensuring satisfactory banding patterns. Finally, the DNA samples meeting the quality requirement were stored at −80 °C in a Hair DW-86L728J freezer for future use. For total RNA extraction from the tissues samples, the procedure strictly adhered to the instructions provided by the Tiangen TRNzol Universal Total RNA Extraction Kit (TIANGEN® DP424). Following RNA extraction, the concentration and purity of the RNA were determined using appropriate methods. Once the RNA samples met the acceptable criteria, including adequate concentration and purity, they were stored at −80 °C in the Hair DW-86L728J freezer to maintain their integrity and stability for subsequent analysis.

#### Library construction and sequencing

The genomic DNA from the samples underwent third-generation long-read sequencing following the standard protocol provided by Oxford Nanopore Technologies (ONT). Initially, the genomic DNA was randomly fragmented using Megaruptor (Diagenode, USA). Subsequently, adapter preparation and ligation were performed using the Nanopore SQK-LSK 109 kit (Oxford Nanopore Technologies, USA). The ligated DNA library was then quantified using the Qubit 3.0 Fluorometer. Finally, the prepared sample was loaded onto Nanopore Flow cells R9.4 and sequenced on the PromethION platform. The sequencing statistics are summarized in Table 1. In total, 577,228 reads were obtained, accumulating a substantial base count of 52,490,712,237 bp. The average read length was 90,935.9 bp, with an N50 length of 100,823 bp. Moreover, the GC content of reads was calculated to be 42.82%.

**Table 1:** Statistics of sequencing data.

Platform	Tissue	Reads	Total bases(bp)	Average length (bp)	N50 length (bp)	GC(%)	GSA accession
PacBioHiFi	blood	4,261,430	71,413,769,333	16,758	16,838	42.61	CRR934932
Ont	blood	577,228	52,490,712,237	90,935.80	100,823	42.82	CRR934933CRR934934
Hic	muscle	1,075,285,592	161,292,838,800	150	150	43.06	CRR934935
RNA-seq	brain	41,033,794	6,155,069,100	150	150	43.31	CRR934940
	heart	45,882,692	6,882,403,800	150	150	47.68	CRR934942
	liver	44,294,376	6,644,156,400	150	150	46.31	CRR934937
	spleen	38,462,044	5,769,306,600	150	150	46.51	CRR934941
	lungs	43,110,100	6,466,515,000	150	150	45.84	CRR934938
	kidney	42,951,176	6,442,676,400	150	150	46.58	CRR934939
Iso-Seq	Mixed	48,373,842	84,725,302,734	1,751.50	2,447	46.05	CRR934943

The samples underwent HiFi sequencing using the Pacific Biosciences single-molecule real-time (SMRT) circular consensus sequencing (CCS) library preparation method. Initially, a total of 100 μg high-quality genomic DNA was sheared into fragments with a target size of approximately 20 kb using Covaris g-TUBEs (Covaris). The size distribution of the sheared genomic DNA was assessed using the Agilent 2100 Bioanalyzer DNA 12000 chip (Agilent Technologies) to ensure they met the required specifications. Subsequently, the PacBio DNA Template Prep Kit 2.0 (Pacific Biosciences of California, Inc., CA) was utilized for constructing sequencing libraries suitable for HiFi sequencing on the PacBio RS II instrument (Pacific Biosciences of California, Inc.). The prepared libraries were then loaded onto a SMRT CELL for sequencing. In total, 4,261,430 reads were generated as sequencing data as summarized in Table 1, with a cumulative base count of 71,413,769,333 bp. The average read length was calculated to be 16,758 bp, with an N50 length of 16,838 bp. Additionally, the GC content of the reads was determined to be 42.61%.

The genomic library for second-generation short-read sequencing of the samples was constructed as follows: Initially, high-quality genomic DNA was randomly sheared using a Covaris sonicator (Covaris, USA). Subsequently, the TruSeq Nano DNA HT Library Preparation Kit (Illumina, USA) was employed to generate an Illumina sequencing library with a target insert size of 350 bp. Finally, the purified library was loaded onto the Illumina NovaSeq 6000 platform for sequencing, which generated a total of 385,826,042 sequences. The cumulative sequencing data obtained from this process amounted to 57,873,906,300 bp. The GC content of the sequences was calculated to be 43.51%.

#### Hi-C sequencing library construction and sequencing

The construction and sequencing of the Hi-C libraries followed a standard protocol with certain modifications^12^. Initially, pectoral muscle tissue samples were cross-linked using a 4% formaldehyde solution at room temperature. Subsequently, the nuclear pellets were re-suspended in 20 μl of lysis buffer, followed by re-suspension of nuclei using 100 μl of NEB buffer. The dissolved nuclei were then lysed using a diluted SDS lysis buffer. DNA was subsequently digested using the four-base cutter MboI, and the DNA ends were labeled using biotin-14-dCTP. Biotin was later removed using T4 DNA polymerase. Followed by DNA ligation using T4 DNA ligase. Finally, after DNA purification, paired-end 150 bp sequencing was conducted on the Illumina HiSeq platform. The sequencing results are summarized in Table 1: A total of 1,075,285,592 reads were obtained, accumulating a substantial base count of 161,292,838,800 bp. The average read length was determined to be 90,935.80 bp, with an N50 length of 100,823 bp. Additionally, the average GC content of the reads was calculated to be 42.82%.

#### RNA Library construction and sequencing

For the construction and sequencing of RNA libraries for the six samples, total RNA was isolated from the brain, heart, liver, spleen, lung, and pectoral muscle tissues using the EasyPure RNA Kit (Transgen). The NEBNext® UltraTM RNA Library Prep Kit for Illumina® (NEB, Ipswich, MA, USA) was then employed to prepare sequencing libraries from the isolated RNA samples. Finally, paired-end (2 × 125 bp) sequencing was performed on the Illumina HiSeq Xten platform. The sequencing results were detailed in Table 1. Among the samples, the heart tissue yielded the highest number of reads, with a total of 45,882,692 reads obtained. Conversely, the spleen tissue had the lowest number of reads with 38,462,044 reads obtained. The average total number of reads across the six tissues was 6,393,354,550 bp, with a calculated GC content of 46.04%.

For the construction and sequencing of full-length transcriptome libraries from mixed samples, we employed the PacBio Sequel system (Pacific Biosciences, CA, USA) for Isoform Sequencing (Iso-Seq). Following the Iso-Seq protocol, cDNA synthesis and amplification were performed using the NEBNext Single Cell/Low Input cDNA Synthesis & Amplification Module. Subsequently, the PacBio SMRTbell Express Template Prep Kit 2.0 was utilized for sample processing, including adapter ligation and addition of SMRTbell sequences. To ensure high-quality and complete transcripts, size-selective purification was conducted using the ProNex® Size-Selective Purification System to remove low-quality and short fragment sequences, completing the preparation of the Iso-Seq library. Finally, high-quality full-length transcriptome sequencing was carried out on the Sequel System (Pacific Biosciences) to obtain accurate and comprehensive full-length transcript sequences. In total, 48,373,842 reads were obtained, resulting in a cumulative data size of 84,725,302,734 bp. The average read length was determined to be 1,751.50 bp, with an N50 length of 2,447 bp. Moreover, the GC content of the reads was calculated to be 46.05%.

### Genome sequence assembly

#### Genome size estimation

In this study, the genome size of the Taihu goose was estimated using the K-mer method based on second-generation short-read sequencing data^13^. The distribution of K-mers was analyzed by examining the paired-end sequencing library data and utilizing the Jellyfish tool^14^. The K-mer distribution was further modeled using GenomeScope (v2.0) to gain preliminary insights into the characteristics of the Taihu goose genome^15^. In Fig. 1C, the observed distribution of K-mers in the sequenced genomic sequences of the Taihu goose is represented by the blue line. Additionally, the brown line represents the K-mers resulting from sequencing errors, which typically have lower frequencies as these errors occur randomly during sequencing. Based on the analysis of the K-mer distribution, GenomeScope was applied to model and estimate the length of the Taihu goose genome to be approximately 1.12 Gb, with a genome heterozygosity of around 0.5%.

#### Genome assembly

In this study, a comprehensive stategy combining Hifiasm (v0.18.5)^16^ and NextDenovo (v2.4.0)^17^ software was employed for genome assembly. Hifiasm was utilized to assemble the genome using various datasets, including Hifi data alone, Hifi + Hi-C data, and Hifi + ONT long reads + Hi-C data. Additionally, NextDenovo was used for assembly using the Hifi + ONT long reads + Hi-C data. The assembly results are summarized in Table 2, where NextDenovo demonstrated superior performance with 244 contigs and an N50 length of 33,928,929 bp, which was selected for downstream analysis. To refine the assembly, the run_purge_dups.py (v1.2.4)^18^ tool was employed to eliminate duplicate contigs. Given the lower accuracy of ONT third-generation long-read sequencing, error correction was performed using Hifi data. This involved counting k-mer occurrences using the meryl software (v1.4)^19^, aligning the assembled genome with the Hifi data using winnowmap software (v2.03)^20^, and subsequent secondary filtering and removal of chimeric alignments using the falconc software (v1.15.0)^21^. Following these steps, three rounds of correction were conducted using the racon software (v1.5.0)^22^ to obtain the final genome assembly sequence after Hifi correction. For high-quality genome assembly and complete scaffold sequences, the Chromap software (v0.2.5)^23^ and yahs (v1.2a.1)^24^ software were utilized in conjunction with Hi-C data. The assembled scaffold sequences were then compared with the known Swan Goose genome (GCA_025388735.1) to identify and align them. This comparison enabled the determination of correspondence between the scaffold sequences and each chromosome of the Swan Goose genome, leading to the renaming of the scaffold sequences based on the matching chromosomes 1–38 and the Z chromosome.

**Table 2:** Statistics of contig assembly before scaffolding.

Statistics without reference	HiFi	HiFi + ONT	HiFi + ONT + Hic
Number of contigs	1136	249	244
Largest contig	35659318	190055209	190055730
Total length	1301139151	1296266443	1296413680
GC(%)	43.57	43.56	43.56
N50	6678817	33928929	33928929
N90	916616	6876214	5566519
L50	54	9	9
L90	265	41	42

**Fig. 2:**
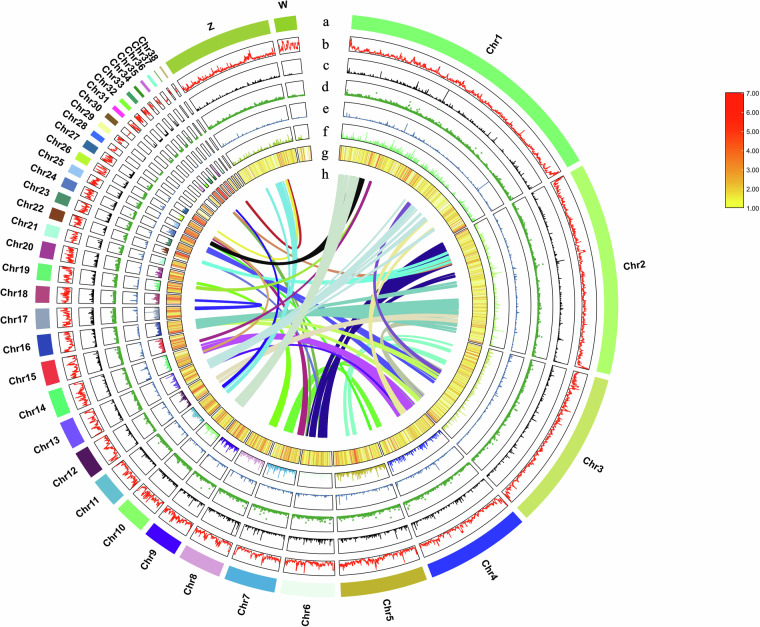
A circos plot of 40 chromosomes of Taihu goose genome assembly. The rings from outside to inside indicate (**a**) chromosomes of the Taihu goose genome, (**b**) GC density, (**c**) exon density, (**d**) CDS density, (**e**) lncRNA density, (**f**) mRNA density, and (**g**) gene density, b-g were drawn in 100 kb; The innermost circle is a collinear plot of homologous genes on different chromosomes.

To obtain more comprehensive sequence information of the goose W chromosome, we conducted additional targeted assembly work specifically focusing on the W chromosome. In the initial published version of the goose genome, the sequence information for the W chromosome was incomplete. Therefore, we employed the W chromosome of a closely related species, the duck, as a reference. By utilizing the “scaffold” module of the ragtag.py software (v2.1.0)^25^, we merged and joined previously unconnected scaffolds to construct the W chromosome of the goose. We assembled a W chromosome with a length of 17.35 Mb. The W chromosome consists of 18 scaffolds, with scaffold_42 representing the primary portion of the W chromosome, accounting for 9.63% of its total length, and the average depth of coverage is 0.996. The detailed coverage results were shown in Table S1. Overall, our efforts resulted in the successful assembly of 38 autosomes, as well as the W and Z sex chromosomes, providing the most complete and comprehensive goose genome assembly achieved to date (Fig. 2). This achievement holds significant academic value for further research on the sex determination mechanism and genetic characteristics of geese.

#### Gap filling

**Fig. 3:**
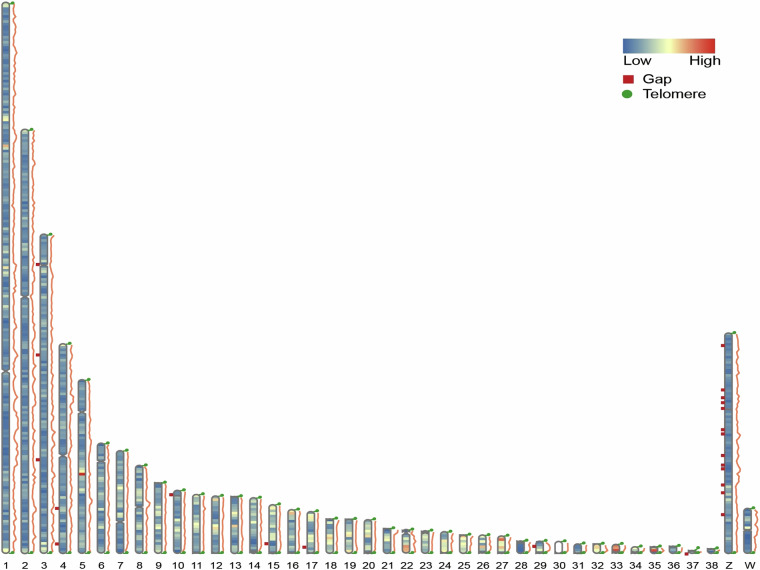
Distribution map of centromeres, telomeres, and gaps on the assembled goose genome. The chromosome heatmap shows the gene density and the wavy lines represent the density of repeat regions.

To address any remaining gaps in the assembled scaffold sequences, we employed the quarTeT software (v1.1.3)^26^ for gap filling. The specific parameters used were “-GapFiller -g *fasta -t 30 -l 5000 -i 60”. The quarTeT tool utilizes quartet alignment information to fill the gaps and enhances the accuracy of the filling process by incorporating additional known genome information. Following the gap filling procedure, we successfully closed all 33 autosomes, with the exception of a few remaining gaps on the two sex chromosomes. The distribution of these gaps across the chromosomes is depicted in Fig. 3.

#### Genome completeness assessment

The Taihu goose genome assembly was subjected to gene structures prediction using the metaeuk software (v6.a5d39d9)^27^. Predicted gene sequences were then compared to a reference dataset of eukaryotic avian species using HMMER (v3.3.2)^28^. By analyzing the alignment and coverage information between the predicted gene sequences and the reference sequences, we assessed the completeness of the Taihu goose genome assembly in terms of conserved gene content. Through statistical analysis of the alignment results, we identified both single-copy genes (S) and multi-copy genes (D) present in the assembled genome. Notably, 96.5% of the single-copy genes aligned completely to the genome. Similarly, 0.4% of the multi-copy genes were found to be entirely present in the genome. To further evaluate the quality of the genome assembly, the Quast software (v5.2.0) was utilized to assess key metrics. The Taihu goose genome size was determined to be 1,197,991,206 bp, with a scaffold N50 of 81,007,908 bp. Compared to previously published chromosome-level goose genomes^4–6^, our assembly exhibited a significant reduction in the number of scaffolds, totalling only 73. Importantly, the scaffold N50 exceeded 80 Mb, signifying a substantial improvement in assembly continuity compared to previous genome versions. Detailed comparative results can be found in Table 3.

**Table 3:** Genome assembly statistics.

Feature	TH	ST	XGG	TFG
Genome size (bp)	1,197,991,206	1,277,289,474	1,163,486,048	1,277,099,016
Number of scaffolds	73	1,266	2242	2055
N50 of scaffolds (bp)	81,007,908	27,064,542	19,834,234	1,849,874
GC content of the genome (%)	42.92	42.39	42.07	42.15
Number of gaps	25	1227	2203	2016
Number of gap-close chromosomes	33	/	/	/
BUSCO completeness (%)	96.9%	96.9%	95.3%	95.2%
Fragmented BUSCOs (%)	0.6	1.1	1	1.1

#### Gene annotation

The RepeatMasker software (v4.1.5)^29^ was utilized to annotate repetitive sequences in the genome of the goose. According to Table 4, dispersed repeats accounted for 8.92% of the total length of the goose genome, with a cumulative length of approximately 106.89 Mb. Among these repeats, retrotransposons constituted about 77.17 Mb (6.44%), while DNA transposons accounted for 3.66 Mb. Notably, Long Interspersed Nuclear Elements (LINEs), comprised around 4.87% of the Taihu goose genome sequences, representing the most abundant type of repetitive sequence. It is worth mentioning that the avian-specific retrotransposon CR1 (Chicken Repeat 1) exhibited the highest abundance, constituting nearly 100% of all LINEs. In addition, Long Terminal Repeats (LTRs) accounted for 1.49% of the Taihu goose genome sequences, while Small Interspersed Nuclear Elements (SINEs) comprised 0.08%. To annotate protein-coding genes and mRNA in the Taihu goose genome, the Liftoff software (v1.6.3)^30^ was employed following the masking of repetitive sequences. The NCBI goose genome reference (GCF_002166845.1), along with its annotation information and transcriptome dataset was used for this purpose. The annotation results revealed a total of 34,898 genes, 436,162 mRNAs, 806,158 Exons, 743,910 Introns, 651,148 CDS and 135,622 UTRs that were annotated in the Taihu goose genome.

**Table 4:** Statistics of repetitive elements.

Class	Subclass	Number	Total length (bp)	% of genome
SINEs		7689	1013259	0.08
LINEs		122541	58287220	4.87
	L2/CR1/Rex	122320	58237288	4.86
	L1/CIN4	93	21924	0.01
LTR elements		28300	17870876	1.49
	Retroviral	28075	17832332	1.49
DNA transposons		22902	3660969	0.31
	hobo-Activator	3590	640205	0.05
	Tc1-IS630-Pogo	630	116362	0.01
	Tourist/Harbinger	6973	698779	0.06
Unclassified		3071	537460	0.04
Total interspersed repeats			81369784	6.79
Small RNA		2810	409311	0.03
Satellites		1368	291347	0.02
Simple repeats		435729	20666486	1.73
Low complexity		73734	4396338	0.37
Total bases masked			106889620	8.92

#### Telomere and centromere identification

In our study, we employed the telomere recognition “TTAGGG,” which is specific to animals, to identify telomere in the goose genome. The TeloExplorer function of the quarTeT software (v1.1.3)^25^ was utilized for telomere identification. The results revealed that chromosome 3 exhibited the highest number of telomere repeat sequences within a 10,000 bp window at each end, with 1,101 and 1,793 repeats, respectively. A visual representation of the telomere distribution can be seen in Fig. 3. To identify centromere, we used the centromics software which is available at https://github.com/ShuaiNIEgithub/Centromics. The assembled genome was analyzed using ont and hifi datasets, along with Hi-C data, to identify the centromeres. By analyzing the peak values in the HiC and TR-CL2 data, we determined the positions of the centromeres on the chromosomes. The locations of the centromeres have been indicated on the chromosome ideogram, as shown in Fig. 3.

## Data Records

The PacBio HiFi, Nanopore, Hi-C and Illumina sequencing data have been deposited in the GSA at CNCB-NGDC CRR934932^31^, CRR934933^32^, CRR934934^33^, CRR934935^34^, and CRR934936^35^ respectively.

The transcriptomic sequencing data of liver, lung, kidney, brain, spleen, heart, and mix-tissue were deposited in the GSA at CNCB-NGDC CRR934937^36^, CRR934938^37^, CRR934939^38^, CRR934940^39^, CRR934941^40^, CRR934942^41^ and CRR934943^42^, respectively.

The T2T genome assembly data have been deposited on GenBank under accession number JBECYW010000000^43^.

## Technical Validation

We employed the 3 C criterion, encompassing Contiguity, Completeness, and Correctness, to comprehensively evaluate the quality of our genome assembly. The resulting chromosome-level genome assembly measured 564.5 Mb, with a scaffold N50 value of 81 Mb. In terms of quantitative assessment, the BUSCO(v5.4.5)^44^ analysis demonstrated the completeness of genomes was 96.90% (Table 3), indicating a highly comprehensive assembly of the goose genome.

The Hi-C heatmap revealed a well-organized pattern of interaction contacts along the diagonals within and around the chromosome inversion region (Fig. 1D), thereby indirectly confirming the accuracy of the chromosome assembly.

### Supplementary information

Supplementary Table S1. The detpth of coverage of W chromsome

## Data Availability

All software and pipelines were executed according to the manual and protocols of the published bioinformatic tools. The version and code/parameters of software have been described in Methods. No custom scripts or code were used.
